# Association between different concentrations of human serum albumin and 28-day mortality in intensive care patients with sepsis: A propensity score matching analysis

**DOI:** 10.3389/fphar.2022.1037893

**Published:** 2022-12-12

**Authors:** Weigan Xu, Jianyang Huo, Guojun Cheng, Juan Fu, Xiangqing Huang, Jinxia Feng, Jun Jiang

**Affiliations:** ^1^ Department of Emergency, First People’s Hospital of Foshan, Foshan, China; ^2^ The Poison Treatment Centre of Foshan, First People’s Hospital of Foshan, Foshan, China

**Keywords:** sepsis, human serum albumin, concentrations, 28-day mortality, MIMIC-IV

## Abstract

**Background:** Human serum albumin (HSA) is a commonly used medication for the treatment of sepsis. However, there is no conclusive evidence as to whether different concentrations of HSA are associated with patient prognosis. This study aimed to evaluate the association between different concentrations of HSA and 28-day mortality in patients with sepsis.

**Methods:** The data for this retrospective study were collected from the Medical Information Mart for Intensive Care IV database. Patients with sepsis were divided into two groups according to the concentration of HSA received: 25% and 5% HSA. The primary outcome of this study was the 28-day mortality in patients with sepsis. To ensure the robustness of our findings, we used multivariate Cox regression, propensity score matching, double-robust estimation, and inverse probability weighting models.

**Results:** A total of 76,943 patients were screened, of whom 5,009 were enrolled. 1,258 and 3,751 patients received 25% and 5% HSA, respectively. The 28-day mortality rate was 38.2% (481/1,258) for patients in the 25% HSA group and 8.7% (325/3,751) for patients in the 5% HSA group. After propensity score matching, 1,648 patients were identified. The inverse probability weighting model suggested that 5% HSA received was associated with lower 28-day mortality (hazard ratio [HR]: 0.63, 95% confidence interval [CI]: 0.54–0.73, *p* < 0.001). Subgroup and sensitivity analysis confirmed the robustness of the results.

**Conclusion:** In patients with sepsis, 5% HSA received may be associated with a lower risk of 28-day mortality than 25% HSA. Further randomized controlled trials are required to confirm this association.

## 1 Introduction

Sepsis is a public health problem characterized by life-threatening organ dysfunction caused by a dysregulated host response to infection (Rhodes et al., 2016). A recent study indicated that the incidence of sepsis in the United States ranges between 30% and 80% each year (Jawad et al., 2012), and at least 1.7 million patients die of sepsis during their hospitalization each year, accounting for more than half of all hospital deaths (Liu et al., 2014). Due to the increased vascular permeability caused by the inflammatory response, sepsis is often combined with clinical features such as edema, hypoalbuminemia, and hypovolemia (Aird, 2003; Czabanka et al., 2007; Schouten et al., 2008). Human serum albumin (HSA) corrects not only hypoalbuminemia but also hypovolemia and ensures tissue perfusion (Jones et al., 2008; Xu et al., 2014), and is a commonly used drug in the clinical treatment of sepsis (Hariri et al., 2018). However, it remains unclear whether the efficacy and safety of different concentrations of HSA solutions are consistent in patients with sepsis.

The 2016 Surviving Sepsis Campaign (SSC) guidelines suggested that HSA may be used as a supplemental resuscitation fluid (Rhodes et al., 2016). The safety profile of HSA in sepsis patients has been demonstrated in several studies but is not associated with patient mortality (Finfer et al., 2004) (Caironi et al., 2014) (Park et al., 2019). In a meta-analysis of randomized controlled trials (RCTs), it was found that HSA infusion may be associated with a reduction in 90-day mortality (Xu et al., 2014). However, several studies have compared mortality in critically ill patients administered different concentrations of HSA, and the results of these studies were not identical (Bannard-Smith et al., 2015; Martensson et al., 2018; O'Brien et al., 2021). Currently, there are no clear recommendations regarding the optimal HSA concentrations for the treatment of patients with sepsis. In critically ill patients, there was little evidence from RCTs or guidelines that mentioned the effect of HSA concentrations on the risk of death in patients with sepsis. Consequently, further research is required to determine the efficacy of infusions with different HSA concentrations in patients with sepsis.

Based on the above studies, we hypothesized that different concentrations of HSA may have different effects on the prognosis of sepsis. In this study, we aimed to compare the difference between 25% and 5% HSA on the 28-day mortality in patients with sepsis. Additionally, we used propensity score matching to minimize potential bias in the baseline characteristics between the 25% and 5% HSA groups. Based on this approach, the results of this study are more reliable.

## 2 Methods

### 2.1 Data sources

The data used in this retrospective study were extracted from the Medical Information Mart for Intensive Care IV (MIMIC-IV) database (Guo et al., 2022), which contains comprehensive information on patients admitted in the Beth Israel Deaconess Medical Center (BIDMC) between 2008 and 2019, including 76,943 adult patients admitted to the intensive care unit (ICU). MIMIC-IV database access was approved by the Massachusetts Institute of Technology (Cambridge, MA, United States) and BIDMC. Consent to participate was obtained during the original data collection (Johnson et al., 2022). The identities of all patients in the database have been removed to protect privacy, and the need for informed consent was waived. One author Weigan Xu was approved to access the database (certification number: 46450588). This study followed the Reporting of Observational Studies in Epidemiology guidelines (von Elm et al., 2014) and regulations (Declaration of Helsinki).

### 2.2 Study population

To screen participants for this study, participants who met the following criteria were included in the study: 1) participants with a diagnosis of sepsis (The diagnosis of sepsis was based on the sepsis 3.0 criteria, which was defined as the earliest time at which a patient has [Sequential Organ Failure Assessment] SOFA ≥2 and suspicion of infection) (Singer et al., 2016). 2) age ≥18 years; 3) who were monitored in the ICU for at least 24 h, and 4) who received 5% or 25% HSA within ICU admission. The diagnosis of sepsis was made within 24 h of ICU admission. Patients who did not receive HSA or who received both 25% and 5% HSA within ICU admission were excluded. In the case of multiple admissions to the ICU, only the data from the first admission were extracted (Chen et al., 2021; Hu et al., 2021).

### 2.3 Drug exposure

HSA use was defined as the documented use of HSA during ICU admission. Patients receiving 5% HSA during ICU stay were categorized as low concentration of HSA group and those receiving 25% HSA were categorized as high concentration of HSA group.

### 2.4 Data extraction

We extracted the data of the following variables from the MIMIC-IV (version 2.0) database on the first day of ICU admission: sex, age, weight, ethnicity, Simplified Acute Physiology Score II (SAPS II), SOFA score, Charlson Comorbidity Index, mean blood pressure (MBP), systolic blood pressure (SBP), diastolic blood pressure (DBP), respiratory rate, heart rate, pulse oximeter oxygen saturation (SPO_2_), temperature, white blood cells (WBC) count, hemoglobin, platelet, blood urea nitrogen (BUN), serum creatinine (Scr), albumin, anion gap, bicarbonate, glucose, sodium, potassium, chloride, prothrombin time (PT), activated partial thrombin time (APTT), lactate, urine output, ventilator use, vasopressor use, renal replacement therapy (RRT) use, and comorbidities (atrial fibrillation, coronary artery disease [CAD], congestive heart failure [CHF], cerebrovascular disease, chronic lung disease, liver disease, diabetes mellitus, renal disease, and malignancy). The average values of laboratory parameters and vital signs within 24 h of ICU admission were used in this study (Hu et al., 2021; Zhang et al., 2021; Wang et al., 2022). We extracted the data related to comorbidities using the International Classification of Diseases (ICD)-9 or ICD-10 diagnosis codes. In addition, we attempted to extract C-reactive protein, troponin t, and B-type natriuretic peptide (BNP) to analyze the inflammatory status and cardiovascular function of the patients. However, we were ultimately unable to include these covariates due to too many missing values for these covariates. All information was extracted from the MIMIC-IV database using the Navicat Premium software (version 16.0). For missing data in continuous variables, we impute with the median or mean of non-missing values.

### 2.5 Primary and secondary study endpoints

The primary study endpoint was 28-day mortality. The secondary study endpoints were in-hospital mortality, length of hospital stay, and length of ICU stay.

### 2.6 Statistical analysis

The patients were divided into two groups according to the concentration of HSA received within ICU admission for descriptive analysis. Normally distributed continuous variables were expressed as mean ± standard deviation (SD). Non-normally distributed data were expressed as medians (interquartile range [IQR]). Categorical variables were expressed as frequencies or percentages. To compare the baseline characteristics of the two groups, t-tests or one-way ANOVA were used for continuous variables, and the chi-square test or Fisher test was used for categorical variables.

Propensity score matching (PSM) and Cox regression analysis were used to balance between-group confounders to reduce the effect of potential bias as much as possible (Shen et al., 2019). PSM included the following matched variables: sex, age, weight, ethnicity, SAPS II score, SOFA score, Charlson Comorbidity Index, MBP, SBP, DBP, respiratory rate, heart rate, SPO_2_, temperature, WBC, hemoglobin, platelet, BUN, Scr, albumin, anion gap, bicarbonate, glucose, sodium, potassium, chloride, PT, APTT, lactate, urine output, vasopressor use, ventilator use, RRT use, and comorbidities. A matched caliper (0.2) was used to match the patients between the two groups, and a standard deviation of 10% was considered sufficient to balance out the distribution between the two groups. Propensity scores were calculated from Cox regression models, along with the hazard ratios (HRs) and 95% confidence intervals (CIs) for each estimate.

Traditionally, when applying regression analysis or propensity score models to estimate causal effects, these methods are unbiased only if both statistical models are correctly specified. The doubly robust estimation approach combines a multivariate regression model with a propensity score model to estimate the association and causal effect of exposure on the outcome (Funk et al., 2011; McCaffrey et al., 2013; Li and Shen, 2020; Zhao et al., 2020; Liu et al., 2022), which can result in an unbiased effect estimate. Therefore, to ensure the accuracy of the results of this study, a double robust estimation approach was used to further confirm the association between 5% HSA and 28-day mortality.

To further validate the robustness of the 5% HSA and the association with 28-day mortality after PSM, an extended Cox regression model approach was used to adjust for different covariates (Yang et al., 2021). Survival analysis was performed using Kaplan-Meier curves and log-rank tests. Statistical analyses in this study were performed using R software (Version 4.0.0) and Free Statistics software (Version 1.5). A two-sided *p* < 0.05 was considered to be statistically significant.

### 2.7 Subgroup analysis and sensitivity analysis

To assess the robustness of the results and how the application of various correlational inference models affects our conclusions, we performed a subgroup analysis of several relevant covariates and described them as forest plots. In addition, we performed two sensitivity analyses. First, patients with liver disease may have hypoproteinemia, which is a common reason for requiring HSA infusion. After excluding patients with liver disease from the data, the first sensitivity analysis was performed. In addition, considering that baseline serum albumin is an important covariate reflecting the nutritional status of the patient. We performed a second sensitivity analysis after excluding patients with missing values of serum albumin.

## 3 Results

### 3.1 Population and baseline characteristics

**FIGURE 1:**
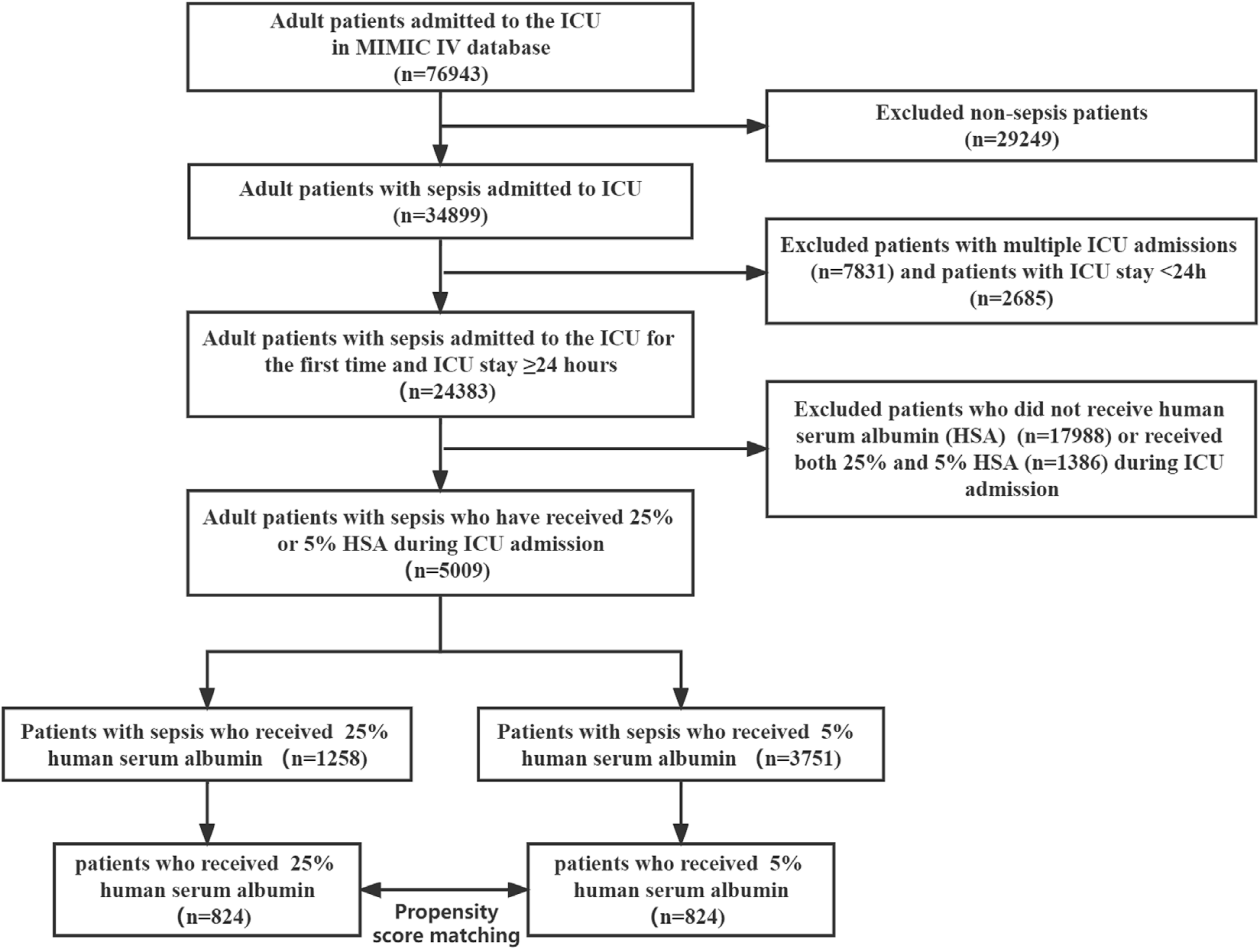
Flowchart of study patient enrollment. ICU, intensive care unit.

The MIMIC-IV database (version 2.0) contains data on 76,943 adult ICU admissions. In the study cohort, we identified 5009 patients with sepsis based on inclusion criteria, of whom 1258 received 25% HSA, while 3751 received 5% HSA (Figure 1). After propensity score matching, 824 pairs of patients were matched and patient characteristics were balanced.

The baseline characteristics of all participants are presented in Table 1. The mean age of the participants was 66.4 years, 1,876 (37.5%) were female, and 2,815 (56.2%) were white individuals. Of 5009 patients, 1258 (25.1%) received 25% HSA and 3751 (74.9%) received 5% HSA. Before PSM, there were statistically significant differences in sex, age, ethnicity, SAPS II, SOFA score, Charlson Comorbidity Index, respiratory rate, heart rate, SPO_2_, hemoglobin, BUN, Scr, albumin, anion gap, bicarbonate, sodium, potassium, chloride, PT, APTT, lactate, urine output, vasopressor use, ventilator use, RRT use, atrial fibrillation, hypertension, CAD, liver disease, renal disease, and malignancy between the two groups. After PSM, 824 pairs matched. The standardized differences of covariates between the 25% HSA group and the 5% HSA group were less than 10%, except for platelets and CAD. For patient disease severity scores, including SAPS II, SOFA score, and Charlson Comorbidity Index, there was no difference between the two groups. There was a tendency that the patients in the 5% HSA group to use more ventilators, vasopressors, and RRT, although it was not statistically significant.

**TABLE 1:** Demographics and baseline characteristics of patients before and after propensity score matching.

Variables	Unmatched Patients			Propensity Score Matched Patients		
	25% HSA (n = 1258)	5% HSA (n = 3751)	SMD	25% HSA (n = 824)	5% HSA (n = 824)	SMD
Sex, female, n (%)	533 (42.4)	1343 (35.8)	0.135	367 (44.5)	385 (46.7)	0.044
Age (years)	62.77 (14.55)	67.65 (13.08)	0.352	65.11 (14.91)	64.74 (14.43)	0.025
Ethnicity, white, n (%)	833 (66.2)	2706 (72.1)	0.111	557 (67.6)	556 (67.5)	0.003
Weight (kg)	86.12 (26.59)	85.69 (20.60)	0.018	84.81 (24.59)	84.10 (23.55)	0.029
SAPS II score	45.80 (14.50)	40.01 (13.50)	0.413	44.35 (14.01)	44.33 (14.90)	0.002
SOFA score	4.91 (2.72)	3.89 (2.11)	0.420	4.33 (2.38)	4.28 (2.43)	0.020
Charlson Comorbidity Index	6.52 (2.90)	5.36 (2.36)	0.440	6.20 (2.84)	5.91 (2.69)	0.006
MBP (mmHg)	54.84 (13.73)	55.39 (10.80)	0.044	54.66 (13.80)	53.91 (13.53)	0.054
SBP (mmHg)	85.07 (15.75)	83.83 (12.56)	0.087	84.76 (15.69)	83.43 (14.31)	0.089
DBP (mmHg)	43.67 (10.79)	43.08 (8.09)	0.062	43.51 (10.59)	43.00 (9.53)	0.051
Respiratory rate (bpm)	29.20 (7.17)	27.31 (6.11)	0.284	29.13 (7.26)	29.35 (6.84)	0.032
Heart rate (bpm)	110.18 (21.86)	102.29 (18.39)	0.390	109.83 (21.65)	111.56 (22.17)	0.079
SpO_2_ (%)	90.86 (7.71)	92.23 (6.79)	0.189	90.90 (7.78)	90.56 (9.53)	0.039
Temperature (^o^C)	37.40 (0.79)	37.45 (0.65)	0.068	37.48 (0.85)	37.56 (0.81)	0.091
WBC (×10^9^/L)	13.50 (8.80)	12.87 (6.63)	0.081	13.69 (8.97)	13.61 (7.93)	0.010
Hemoglobin (g/L)	9.97 (2.36)	10.34 (2.28)	0.159	10.30 (2.43)	10.49 (2.40)	0.079
Platelet (×10^12^/L)	179.93 (139.25)	186.45 (100.59)	0.054	198.25 (137.98)	217.92 (136.12)	0.144
BUN (mmol/L)	36.66 (28.70)	21.46 (15.00)	0.664	29.77 (21.74)	29.52 (23.58)	0.011
Scr (mg/dl)	1.83 (1.51)	1.15 (0.98)	0.534	1.57 (1.34)	1.58 (1.46)	0.004
Albumin	2.87 (0.61)	2.94 (0.38)	0.142	2.86 (0.56)	2.83 (0.50)	0.068
Anion gap (mEq/L)	16.91 (5.78)	13.40 (4.28)	0.689	15.87 (5.06)	16.16 (5.58)	0.055
Bicarbonate (mmol/L)	21.16 (5.49)	22.61 (3.65)	0.311	21.69 (5.18)	21.33 (5.03)	0.071
Glucose (mg/dl)	148.54 (176.41)	135.45 (60.93)	0.099	147.14 (73.47)	152.42 (92.41)	0.063
Sodium (mmol/L)	136.25 (6.84)	138.76 (4.07)	0.446	137.60 (6.03)	137.56 (5.64)	0.006
Potassium (mmol/L)	4.37 (0.92)	4.25 (0.66)	0.143	4.32 (0.86)	4.32 (0.84)	0.006
Chloride (mmol/L)	101.50 (8.39)	106.61 (5.94)	0.704	103.49 (7.44)	103.35 (7.01)	0.020
PT s)	20.83 (12.75)	16.18 (8.00)	0.438	19.00 (12.14)	18.24 (13.83)	0.059
APTT s)	42.69 (23.27)	38.24 (22.18)	0.196	40.86 (24.05)	39.14 (22.83)	0.073
Lactate (umol/L)	2.98 (2.60)	1.96 (1.75)	0.461	2.64 (2.21)	2.78 (2.64)	0.057
Urine output (ml/24 h)	1331 (1068)	1844 (1216)	0.448	1470 (1096)	1427 (1166)	0.037
Vasopressor use, n (%)	669 (53.2)	2919 (77.8)	0.537	500 (60.7)	515 (62.5)	0.037
Ventilator use, n (%)	679 (54.0)	2591 (69.1)	0.314	500 (60.7)	514 (62.4)	0.035
RRT use, n (%)	108 (8.6)	115 (3.1)	0.237	58 (7.0)	62 (7.5)	0.019
Atrial fibrillation, n (%)	344 (27.3)	1562 (41.6)	0.304	279 (33.9)	253 (30.7)	0.068
Hypertension, n (%)	646 (51.4)	2520 (67.2)	0.326	482 (58.5)	472 (57.3)	0.025
CAD, n (%)	305 (24.2)	2109 (56.2)	0.690	259 (31.4)	217 (26.3)	0.113
CHF, n (%)	291 (23.1)	979 (26.1)	0.069	223 (27.1)	211 (25.6)	0.033
Cerebrovascular disease, n (%)	130 (10.3)	410 (10.9)	0.019	109 (13.2)	102 (12.4)	0.025
Chronic lung disease, n (%)	307 (24.4)	920 (24.5)	0.003	220 (26.7)	205 (24.9)	0.042
Liver disease, n (%)	691 (54.9)	335 (8.9)	1.134	285 (34.6)	259 (31.4)	0.067
Diabetes mellitus, n (%)	345 (27.4)	1104 (29.4)	0.045	233 (28.3)	218 (26.5)	0.041
Renal disease, n (%)	262 (20.8)	601 (16.0)	0.124	166 (20.1)	140 (17.0)	0.081
Malignancy, n (%)	225 (17.9)	297 (7.9)	0.301	141 (17.1)	144 (17.5)	0.010

Abbreviations: HSA, human serum albumin; SAPS II, Simplified acute physiology score II; SOFA, sequential organ failure assessment; MBP, mean blood pressure; SBP, systolic blood pressure; DBP, diastolic blood pressure; WBC, white blood cell; BUN, blood urea nitrogen; Scr, Serum creatinine; PT, prothrombin time; APTT, activated partial thrombin time; RRT, renal replacement therapy; CAD, coronary artery disease; CHF, congestive heart failure.

### 3.2 Primary and secondary study endpoints

The overall 28-day mortality was 16.1% (806/5,009). The 28-day mortality in the 5% HSA group was 8.7% (325/3,751), compared with 38.2% (481/1,258) for the 25% HSA group (Table 2). Compared with patients who received 25% HSA, patients who received 5% HSA had a lower risk of 28-day mortality in the unadjusted model (HR: 0.19, 95% CI: 0.17-0.22, *p* < 0.001). After adjusting for confounding factors, the HR for 5% HSA group in the Cox proportional hazards regression was 0.52 (95% CI, 0.44-0.62, *p* < 0.001) (Table 3).

**TABLE 2:** Study endpoints of 25%HSA and 5% HSA groups before and after propensity score matching.

Variables	Unmatched Patients			Propensity Score Matched Patients		
	25% HSA (n = 1258)	5% HSA (n = 3751)	*p-value*	25% HSA (n = 824)	5% HSA (n = 824)	*p-value*
Primary outcome						
28-day mortality, n (%)	481 (38.2)	325 (8.7)	<0.001	248 (30.1)	191 (23.2)	0.001
Secondary outcomes						
In-hospital mortality, n (%)	438 (34.8)	295 (7.9)	<0.001	233 (28.3)	180 (21.8)	0.003
Los hospital (day)	14.1 (7.8, 24.0)	8.3 (5.4, 14.1)	<0.001	14.5 (8.0, 24.2)	12.7 (6.6, 21.7)	0.001
Los ICU (day)	5.2 (2.8, 11.2)	2.9 (1.5, 5.4)	<0.001	5.9 (3.0, 12.9)	4.0 (2.1, 8.1)	<0.001

Abbreviations: Los, length of stay.

**TABLE 3:** Associations between 5%HSA and the 28-day mortality.

Analysis	28-day mortality (%)	*p-value*
No. of events/no. of patients at risk (%)		
25% HSA	481/1258 (38.2)	
5% HSA	325/3751 (8.7)	
Crude analysis - hazard ratio (95% CI)	0.19 (0.17–0.22)	<0.001
Multivariable analysis - hazard ratio (95% CI)^a^	0.52 (0.44–0.62)	<0.001
Adjusted for propensity Score^b^	0.59 (0.49–0.70)	0.004
With matching^c^	0.76 (0.63–0.91)	<0.001
With inverse probability weighting^d^	0.63 (0.54–0.73)	<0.001
Doubly robust analysis	0.74 (0.60–0.90)	0.002

^a^ Shown is the hazard ratio from the multivariable Cox regression model adjusted for all covariates in table 1.

^b^ Shown is the hazard ratio from a multivariable Cox regression model with the same strata and covariates with additional adjustment for the propensity score.

^c^ Shown is the hazard ratio from a multivariable Cox regression model with the same strata and covariates with matching according to the propensity score. The analysis included 1650 patients (825 who Received 5% human serum albumin and 825 who Received 5% human serum albumin).

^d^ Shown is the primary analysis with a hazard ratio from the multivariable Cox regression model with the same strata and covariates with inverse probability weighting according to the propensity score.

**FIGURE 2:**
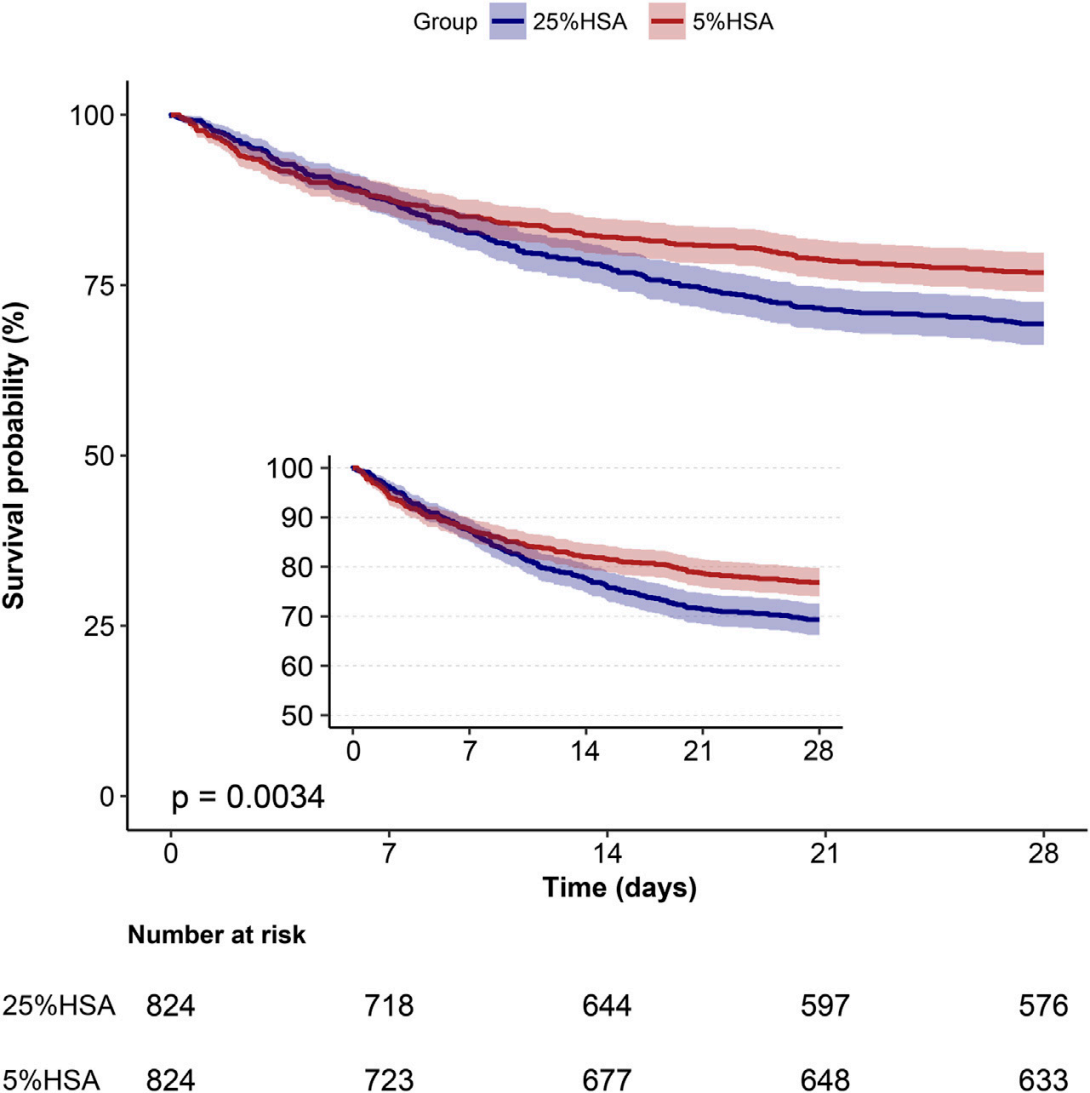
Kaplan-Meier survival curve of 28-day mortality for patients with sepsis.

After PSM, the 28-day mortality (23.2% vs. 30.1%, *p* = 0.001) and in-hospital mortality (21.8% vs. 28.3%, *p* = 0.003) were still lower in the 5% HSA group than in the 25% HSA group, and patients in the 5% HSA group had shorter lengths of hospital stay (12.7% vs. 14.5%, *p* = 0.001) and ICU stay (4.0% vs. 5.9%, *p* < 0.001) (Table 2). The results of the PSM (HR: 0.59, 95% CI: 0.49-0.70, *p* =0.004), IPTW (HR:0.63, 95% CI: 0.54-0.73, *p* < 0.001) and double robust analysis (HR:0.74, 95% CI: 0.60-0.90, *p* < 0.001) models all demonstrated that 5% HSA group had lower 28-day mortality (Table 3). After the univariate Cox regression analysis (Supplementary Table S1), extended multivariate models were used to analyze the data after PSM. As shown in Table 4, the unadjusted HR was 0.76 (95% CI: 0.63–0.91). After adjusting for all confounders, the HR was 0.73(95% CI: 0.60–0.89), and the results remained robust. The Kaplan-Meier curve showed lower 28-day mortality among patients with 5% HSA (log-rank test: *p* = 0.0034) (Figure 2).

**TABLE 4:** Multivariate Cox regression for 5% HSA on 28-day mortality of patients in matched cohort.

	Non-adjusted	Model I	Model II	Model III
Variable	HR (95% CI)	HR (95% CI)	HR (95% CI)	HR (95% CI)
25% HSA	1(Ref)	1(Ref)	1(Ref)	1(Ref)
5% HSA	0.76 (0.63–0.91)	0.76 (0.63–0.92)	0.79 (0.65–0.96)	0.73 (0.60–0.89)

Notes.

Model I: Adjusts for sex + age.

Model II: Adjusts for Model I + weight + ethnicity + SAPS II score + SOFA score + Charlson Comorbidity Index + atrial fibrillation + hypertension + CAD + CHF + cerebrovascular + chronic lung disease + liver disease + diabetes mellitus + renal disease + malignancy + heart rate + respiratory rate + temperature + SpO_2_ +MBP + SBP + DBP.

Model III: Adjusts for Model II + platelet + BUN + Scr + albumin + anion gap + bicarbonate + potassium + chloride + PT + APTT + lactate + urine output + ventilator use + vasopressor use + RRT use.

Abbreviations: SAPS II, Simplified acute physiology score II; SOFA, sequential organ failure assessment; CAD, coronary artery disease; CHF, congestive heart failure; MBP, mean blood pressure; SBP, systolic pressure; DBP, diastolic blood pressure; BUN, blood urea nitrogen; Scr, serum creatinine; PT, prothrombin time; APTT, activated partial thrombin time; RRT, renal replacement therapy.

### 3.3 Subgroup analysis and sensitivity analysis

**FIGURE 3:**
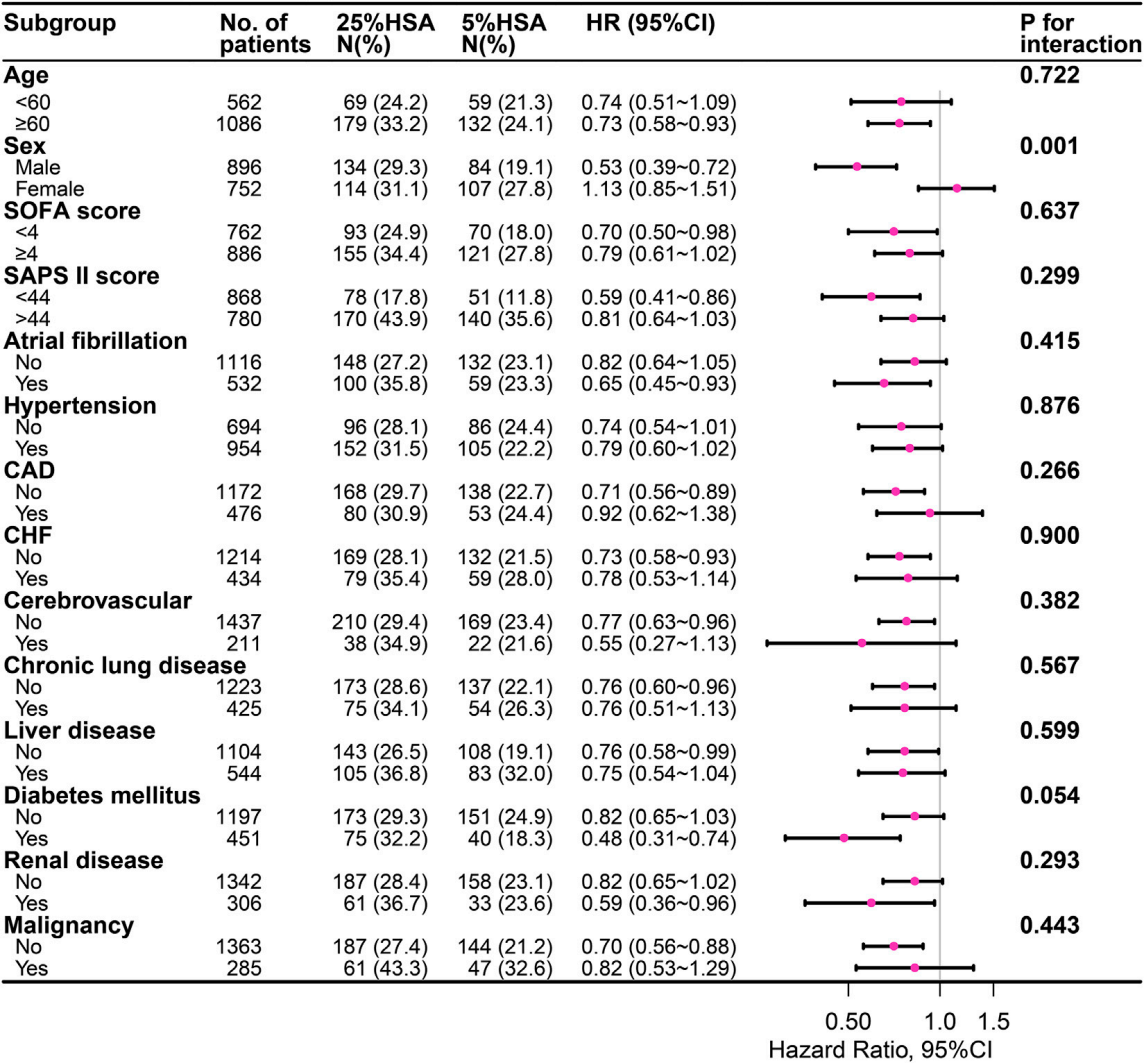
The association between different concentrations of HSA and 28-day mortality in subgroups. HSA, human serum albumin; SOFA, Sequential organ failure assessment; SAPS II, Simplified acute physiology score II; CAD, coronary artery disease; CHF, congestive heart failure.

The subgroup analysis indicated a negative association between 5% HSA and 28-day mortality (Figure 3). In the subgroup analysis, no significant interactions were observed in most subgroups. However, an interaction was found between sex and HSA concentrations (interaction at *p* = 0.001).

In the full cohort (*n* = 5,009), after excluding 1,026 patients with liver disease, 3,983 patients remained. We performed multiple analyses of the data using doubly robust models, IPW models, propensity score matching models, and Cox regression based multivariate analysis models. The results demonstrated that the association between 5% HSA and 28-day mortality was robust (Supplementary Table S2). In addition, serum albumin data were missing for 3,246 patients. After excluding the patients with missing data, we performed the analysis again on the remaining 1,763 patients. The results showed that the association remained robust (SupplementaryTable S3). All sensitivity analyses were consistent with our main finding that 5% HSA was associated with lower 28-day mortality in patients with sepsis.

## 4 Discussion

To our knowledge, this study is a large cohort study on the association of different concentrations of HSA with 28-day mortality in patients with sepsis. In the study, patients receiving 5% HSA had lower risk-adjusted 28-day mortality in sepsis compared to patients receiving 25% HSA.

Multiple large randomized controlled trials, including the SAFE, ALBIOS, and CRISTAL trials, have compared 4% HSA to normal saline solution, 20% HSA to crystals, and a variety of colloids to crystals (Finfer et al., 2004; Annane et al., 2013; Caironi et al., 2014). However, none of these studies investigated the differences between low (4%–5%) and high (20%–25%) concentrations of HSA. Conclusions regarding the efficacy and safety of different concentrations of HSA in patients with sepsis remain controversial. In this study, we investigated the association between different concentrations of HSA (25% vs. 5%) and 28-day mortality in patients with sepsis for the first time.

Similar to our results, a previous meta-analysis included 58 randomized controlled trials involving 26,351 patients, 14,659 of whom had sepsis. The results demonstrated that low concentrations of HSA may reduce mortality compared with high concentrations of HSA, OR = 0.90 (95% CI 0.68–1.18) (Tseng et al., 2020). However, for the confidence interval, this difference was not significant. Schortgen et al. (2008) found that receiving high concentrations of HSA was significantly associated with poor prognosis and increased incidence of AKI in septic shock after adjusting for potential confounders and using propensity score analysis. Bannard-Smith et al. (2015) reported that patients receiving 4% HSA had lower ICU mortality than those receiving 20% HSA (6.9% vs. 18.8%, *p* = 0.01). However, this study only enrolled 202 ICU patients receiving HSA, and this effect was not significant after adjustment for APACHE III (OR: 1.21; 95% CI, 0.69-5.21; *p* = 0.22). In another RCT, the SWIPE trial randomly assigned low (4%–5% HSA) and high (20% HSA) concentrations to 321 ICU patients, including medical and surgical patients (Martensson et al., 2018). The SWIPE trial reported a lower mortality rate in ICU patients who received 20% albumin. However, the authors of the study suggested that this finding may represent a type I error. Owing to imbalances in some baseline characteristics, the adjusted analysis had no significant effect on ICU mortality, and there were only small differences in in-hospital mortality between the groups. Therefore, the results of this study regarding ICU mortality should be interpreted with caution. Additionally, since this study only included 36 patients with sepsis at baseline, it may not be possible to draw reliable conclusions regarding its use in this patient population. Compared to this study, our study had a larger cohort (*n* = 5,009). After adjusting for potential confounders using multiple methods, a stable association was found between receiving 5% HSA and decreased 28-day mortality.

A randomized clinical trial is the best way to investigate whether there is an association between any therapeutic intervention and outcome because it maximizes two major problems in observational studies: unmeasurable confounding and bias. In this observational cohort study, we used multiple methods to try to minimize possible confounding, including PSM, double robust estimation, inverse probability weighting analysis and extended Cox regression models. The primary analysis demonstrated a significant association between 5% HSA received and decreased 28-day mortality in patients with sepsis. The results of the other two sensitivity analyses were similar. Notably, we found a significant interaction by sex in further subgroup analyses. A decrease of 28-day mortality with 5% HSA with sepsis was observed only in men. However, the result should be interpreted with caution due to the small number of cases and wide confidence intervals.

It is still unclear the mechanism by which different concentrations of HSA are associated with reduced 28-day mortality in patients with sepsis. The following issues may need to be considered. First, different concentrations of HSA may have different effects on patients with sepsis. Low concentrations of HSA accumulate more fluid during fluid resuscitation in sepsis (Martensson et al., 2018), and a positive fluid balance is associated with an increased risk of death (Acheampong and Vincent, 2015). High concentrations of HSA, as a highly osmotic solution, may reduce the glomerular net filtration pressure and glomerular filtration rate (GFR), resulting in exacerbated renal dysfunction (Boer et al., 1987; Wiedermann et al., 2010). Second, HSA increases plasma and interstitial compartment volume compared to saline, but at the cost of further intracellular dehydration (Ernest et al., 2001). Any benefits of plasma volume expansion may be offset by intracellular dehydration, and these effects may be more significant when the HSA concentration is higher (Udeh et al., 2018). Third, the properties of oxidative stress may be dissimilar for different concentrations of HSA. Low concentrations of HSA (4%) have anti-inflammatory and antioxidant properties, but high concentrations (20%) have pro-oxidant effects that may be detrimental to endothelial function and organ dysfunction (Kremer et al., 2011). Fourth, different HSA concentrations may not be suitable for the same population. The results of a recent meta-analysis suggested that isotonic albumin is associated with a better survival benefit in septic patients experiencing hypovolemia due to extravascular fluid loss as a result of increased vascular permeability. However, hypertonic albumin has better survival potential in patients undergoing surgery for uncorrected hemorrhage (Ernest et al., 1999).

This study has several limitations. First, owing to the retrospective design, there were unmeasurable confounding factors, such as the etiology of sepsis, antibiotic therapy, and clinician preference. We adjusted for baseline characteristics between groups by PSM and further investigated the primary study endpoint with multiple subgroup analyses to minimize the bias in the results caused by confounding factors. Second, we were unable to review the appropriateness of the indication for infusion of HSA since the basis for the physician’s decision was not documented. However, we believe that our study population met the true clinical profile of HSA infusion in patients with sepsis based on inclusion and exclusion criteria. Third, in the MIMIC-IV database, we were unable to accurately identify whether septic shock was present on the day of ICU admission and may not accurately reflect the severity of the patient’s condition. However, the SAPS II score, SOFA score, and vasopressor use on the first day of ICU admission were included in our study. Finally, our results suggest that 5% HSA is associated with decreased 28-day mortality in sepsis. These findings are hypothesis-generating and should be considered exploratory. We believe that a carefully designed, multicenter prospective study is needed to validate our results.

## 5 Conclusion

Compared to those receiving 25% HSA, patients with sepsis receiving 5% HSA had lower 28-day mortality. Because our study was retrospective, the findings are preliminary, and a prospective randomized controlled study is needed to obtain greater validity.

## Data Availability

Publicly available datasets were analyzed in this study. This data can be found here: STATEMENT: Publicly available datasets were analyzed in this study. This data can be found here: https://physionet.org/content/mimiciv.

## Abbreviations

MIMIC, medical information mart for intensive care; BIDMC, Beth Israel Deaconess Medical Center; PSM, propensity score matching; HSA, human serum albumin; SAPS II, Simplified Acute Physiology Score II; SOFA, Sequential Organ Failure Assessment; MBP, mean blood pressure; SBP, systolic blood pressure; DBP, diastolic blood pressure; WBC, white blood cell; BUN, blood urea nitrogen; Scr, serum creatinine; PT, prothrombin time; APTT, activated partial thrombin time; RRT, renal replacement therapy; CAD, coronary artery disease; CHF, chronic heart failure.
