# Supplementary material for: Association between different concentrations of human serum albumin and 28-day mortality in intensive care patients with sepsis: A propensity score matching analysis
Source: Front Pharmacol. 2022 Dec 12;13:1037893. doi: 10.3389/fphar.2022.1037893 (PMC9792095; doi:10.3389/fphar.2022.1037893)
Supplement: Supplementary file 1 [file Table1.DOCX]

| **Table S1 Univariable Cox regression analysis of risk factors associated with 28-day mortality in patients with sepsis before and after propensity score matching.** | | | | | |
| --- | --- | --- | --- | --- | --- |
| **Characteristic** | **Unmatched Patients** | |  | **Propensity-Score–Matched Patients** | |
|  | HR (95% CI) | *P -value* |  | HR (95% CI) | *P -value* |
| HSA | 0.19 (0.17~0.22) | < 0.001 |  | 0.76 (0.63~0.91) | 0.004 |
| Sex | 1.43 (1.25~1.64) | < 0.001 |  | 1.25 (1.04~1.51) | 0.018 |
| Age | 1.00 (0.99~1.00) | 0.317 |  | 1.02 (1.01~1.02) | < 0.001 |
| Ethnicity | 0.63 (0.55~0.73) | < 0.001 |  | 0.75 (0.62~0.91) | 0.004 |
| Weight | 1.00 (0.99~1.01) | 0.007 |  | 1.00 (0.99~1.00) | < 0.001 |
| SAPS II | 1.05 (1.05~1.06) | < 0.001 |  | 1.04 (1.03~1.05) | < 0.001 |
| SOFA score | 1.18 (1.15~1.21) | < 0.001 |  | 1.09 (1.05~1.12) | < 0.001 |
| Charlson Comorbidity Index | 1.23 (1.20~1.26) | < 0.001 |  | 1.17 (1.14~1.21) | < 0.001 |
| MBP | 0.98 (0.97~0.98) | < 0.001 |  | 0.99 (0.98~0.99) | < 0.001 |
| SBP | 0.98 (0.98~0.99) | < 0.001 |  | 0.98 (0.98~0.99) | < 0.001 |
| DBP | 0.98 (0.97~0.99) | < 0.001 |  | 0.98 (0.97~0.99) | 0.001 |
| Respiratory rate | 1.05 (1.04~1.06) | < 0.001 |  | 1.03 (1.02~1.04) | < 0.001 |
| Heart rate | 1.02 (1.02~1.02) | < 0.001 |  | 1.01 (1.00~1.01) | < 0.001 |
| SpO_2_ | 0.97 (0.96~0.97) | < 0.001 |  | 0.98 (0.97~0.99) | < 0.001 |
| Temperature | 0.79 (0.71~0.88) | < 0.001 |  | 0.78 (0.7~0.87) | < 0.001 |
| WBC | 1.02 (1.01~1.03) | < 0.001 |  | 1.01 (1.00~1.02) | 0.045 |
| Hemoglobin | 0.98 (0.95~1.01) | 0.182 |  | 0.98 (0.94~1.02) | 0.278 |
| Platelet | 1.00 (1.00~1.00) | 0.833 |  | 1.00 (1.00~1.00) | 0.022 |
| BUN | 1.02 (1.02~1.02) | < 0.001 |  | 1.01 (1.01~1.01) | < 0.001 |
| Scr | 1.29 (1.25~1.33) | < 0.001 |  | 1.11 (1.05~1.17) | < 0.001 |
| Albumin | 0.46 (0.40~0.54) | < 0.001 |  | 0.58 (0.49~0.69) | < 0.001 |
| Anion gap | 1.12 (1.11~1.13) | < 0.001 |  | 1.06 (1.04~1.08) | < 0.001 |
| Bicarbonate | 0.90 (0.89~0.91) | < 0.001 |  | 0.96 (0.94~0.97) | < 0.001 |
| Glucose | 1.00 (1.00~1.00) | 0.119 |  | 1.00 (1.00~1.00) | 0.468 |
| sodium | 0.94 (0.93~0.95) | < 0.001 |  | 0.99 (0.97~1.01) | 0.191 |
| potassium | 1.31 (1.20~1.42) | < 0.001 |  | 1.20 (1.08~1.33) | < 0.001 |
| chloride | 0.93 (0.92~0.93) | < 0.001 |  | 0.97 (0.96~0.99) | < 0.001 |
| PT | 1.02 (1.02~1.03) | < 0.001 |  | 1.01 (1.01~1.02) | < 0.001 |
| APTT | 1.01 (1.01~1.01) | < 0.001 |  | 1.00 (1.00~1.01) | < 0.001 |
| Lactate | 1.21 (1.19~1.23) | < 0.001 |  | 1.14 (1.11~1.16) | < 0.001 |
| Urine output | 1.00 (1.00~1.00) | < 0.001 |  | 1.00 (1.00~1.00) | < 0.001 |
| Vasopressor use | 0.65 (0.57~0.75) | < 0.001 |  | 1.07 (0.88~1.30) | 0.489 |
| Ventilator use | 0.80 (0.70~0.93) | 0.002 |  | 1.31 (1.08~1.60) | 0.007 |
| RRT use | 3.83 (3.11~4.73) | < 0.001 |  | 2.36 (1.79~3.10) | < 0.001 |
| Atrial fibrillation | 0.75 (0.65~0.87) | < 0.001 |  | 1.19 (0.98~1.45) | 0.077 |
| Hypertension | 0.65 (0.57~0.75) | < 0.001 |  | 1.03 (0.85~1.24) | 0.788 |
| CAD | 0.40 (0.34~0.47) | < 0.001 |  | 1.09 (0.89~1.33) | 0.431 |
| CHF | 1.10 (0.94~1.29) | 0.223 |  | 1.32 (1.08~1.62) | 0.006 |
| Cerebrovascular disease | 1.17 (0.95~1.45) | 0.137 |  | 1.07 (0.82~1.41) | 0.607 |
| Chronic lung disease | 1.06 (0.90~1.24) | 0.478 |  | 1.23 (1.00~1.51) | 0.050 |
| Liver disease | 4.92 (4.28~5.65) | < 0.001 |  | 1.64 (1.36~1.98) | < 0.001 |
| Diabetes mellitus | 0.76 (0.65~0.90) | < 0.001 |  | 0.92 (0.74~1.13) | 0.421 |
| Renal disease | 1.34 (1.13~1.59) | < 0.001 |  | 1.21 (0.96~1.52) | 0.102 |
| Malignancy | 2.59 (2.19~3.06) | < 0.001 |  | 1.68 (1.35~2.09) | < 0.001 |
| **Abbreviations:** HSA, Human serum albumin; SAPS II, Simplified acute physiology score II; SOFA, Sequential organ failure assessment; MBP, mean blood pressure; SBP, systolic blood pressure; DBP, [diastolic blood pressure](javascript:;); WBC, white blood cell; BUN, [blood urea nitrogen](javascript:;); Scr, serum creatinine; PT, prothrombin time; APTT, activated partial thrombin time; RRT, renal replacement therapy; CAD, [coronary artery disease](https://www.cdc.gov/heartdisease/coronary_ad.htm); CHF, congestive heart failure. | | | | | |

| **Table S2 Sensitivity analysis after removing patients with liver disease.** | | |
| --- | --- | --- |
| Analysis | 28-dayl mortality (%) | *P-value* |
| Crude analysis - hazard ratio (95% CI) | 0.12 (0.10~0.14) | <0.001 |
| Multivariable analysis - hazard ratio (95% CI) ^a^ | 0.41 (0.31~0.53) | <0.001 |
| Adjusted for propensity Score ^b^ | 0.43 (0.32~0.57) | <0.001 |
| With matching ^c^ | 0.60 (0.44~0.81) | <0.001 |
| With inverse probability weighting ^d^ | 0.40 (0.33~0.49) | <0.001 |
| Doubly robust analysis | 0.40 (0.31~0.52) | <0.001 |
| 1. Shown is the hazard ratio from the multivariable Cox regression model adjusted for all covariates in table 1.   b. Shown is the hazard ratio from a multivariable Cox regression model with the same strata and covariates with additional adjustment for the propensity score.  c. Shown is the hazard ratio from a multivariable Cox regression model with the same strata and covariates with matching according to the propensity score.  d. Shown is the primary analysis with a hazard ratio from the multivariable Cox regression model with the same strata and covariates with inverse probability weighting according to the propensity score. | | |

| **Table S3 Sensitivity analysis after removing patients with missing values of serum albumin.** | | |
| --- | --- | --- |
| Analysis | 28-day mortality (%) | *P-value* |
| Crude analysis - hazard ratio (95% CI) | 0.42 (0.35~0.50) | < 0.001 |
| Multivariable analysis - hazard ratio (95% CI) ^a^ | 0.56 (0.46~0.69) | < 0.001 |
| Adjusted for propensity Score ^b^ | 0.63 (0.51~0.77) | < 0.001 |
| With matching ^c^ | 0.74 (0.59~0.93) | 0.009 |
| With inverse probability weighting ^d^ | 0.63 (0.53~0.75) | < 0.001 |
| Doubly robust analysis | 0.56 (0.46~0.68) | <0.001 |
| 1. Shown is the hazard ratio from the multivariable Cox regression model adjusted for all covariates in table 1.   b. Shown is the hazard ratio from a multivariable Cox regression model with the same strata and covariates with additional adjustment for the propensity score.  c. Shown is the hazard ratio from a multivariable Cox regression model with the same strata and covariates with matching according to the propensity score.  d. Shown is the primary analysis with a hazard ratio from the multivariable Cox regression model with the same strata and covariates with inverse probability weighting according to the propensity score.with the same strata and covariates with inverse probability weighting according to the propensity score. | | |
